# Prospective observational pilot study of the T2Resistance panel in the T2Dx system for detection of resistance genes in bacterial bloodstream infections

**DOI:** 10.1128/jcm.01296-23

**Published:** 2024-03-08

**Authors:** Thomas J. Walsh, Antonella Mencacci, Riccardo Paggi, Evangelia Douka, Charikleia Vrettou, Roger Smith, Oscar Guzman

**Affiliations:** 1 Center for Innovative Therapeutics and Diagnostics, Richmond, Virginia, USA; 2 Departments of Medicine and Microbiology & Immunology, University of Maryland School of Medicine, Baltimore, Maryland, USA; 3 Microbiology and Clinical Microbiology, Department of Medicine and Surgery, University of Perugia, Perugia, Italy; 4 Microbiology Unit, Perugia General Hospital, Perugia, Italy; 5 First Department of Critical Care, University of Athens, Evangelismos General Hospital, Athens, Greece; 6 T2 Biosystems, Lexington, Massachusetts, USA; Cleveland Clinic, Cleveland, Ohio, USA

**Keywords:** T2, rapid molecular diagnosis, sepsis, resistance genes, blood cultures

## Abstract

Early initiation of antimicrobial therapy targeting resistant bacterial pathogens causing sepsis and bloodstream infections (BSIs) is critical for a successful outcome. The T2Resistance Panel (T2R) detects the following resistance genes within organisms that commonly cause BSIs directly from patient blood samples: *bla_KPC_*, *bla_CTXM-14/15_*, *bla_NDM_/bla_/IMP_/bla_VIM_*, *bla_AmpC_*, *bla_OXA_*, *vanA*, *vanB*, and *mec*A/*mec*C. We conducted a prospective study in two major medical centers for the detection of circulating resistance genes by T2R in patients with BSIs. T2R reports were compared to antimicrobial susceptibility testing (AST), phenotypic identification, and standard molecular detection assays. Among 59 enrolled patients, 25 resistance genes were identified: *bla_KPC_* (*n* = 10), *bla_NDM_/bla_/IMP_/bla_VIM_* (*n* = 5), *bla_CTXM-14/15_* (*n* = 4), *bla_AmpC_* (*n* = 2), and *mec*A/*mec*C (*n* = 4). Median time-to-positive-T2R in both hospitals was 4.4 hours [interquartile range (IQR): 3.65–4.97 hours] in comparison to that for positive blood cultures with final reporting of AST of 58.34 h (IQR: 45.51–111.2 hours; *P* < 0.0001). The sensitivity of T2R to detect the following genes in comparison to AST was 100% for *bla*_CTXM-14/15_, *bla*_NDM_*/bla_/_*_IMP_*/bla*_VIM_, *bla*_AmpC_, *mec*A/*mec*C and 87.5% for bla_KPC_. When monitored for the impact of significant antimicrobial changes, there were 32 events of discontinuation of unnecessary antibiotics and 17 events of escalation of antibiotics, including initiation of ceftazidime/avibactam in six patients in response to positive T2R results for *bla_KPC_*. In summary, T2R markers were highly sensitive for the detection of drug resistance genes in patients with bacterial BSIs, when compared with standard molecular resistance detection systems and phenotypic identification assays while significantly reducing by approximately 90% the time to detection of resistance compared to standard methodology and impacting clinical decisions for antimicrobial therapy.

**IMPORTANCE:**

This is the first reported study to our knowledge to identify key bacterial resistance genes directly from the bloodstream within 3 to 5 hours in patients with bloodstream infections and sepsis. The study further demonstrated a direct effect in modifying initial empirical antibacterial therapy in response to T2R signal to treat resistant bacteria causing bloodstream infections and sepsis.

## INTRODUCTION

Resistant bacterial pathogens pose a global public health threat to hospitalized patients (1–6). Timely initiation of antimicrobial therapy targeting these resistant organisms in patients with bloodstream infections (BSIs) is critical for the successful outcome of sepsis.

The T2Bacteria Panel was the first licensed rapid molecular diagnostic platform to identify five major bacterial pathogens (*Staphylococcus aureus*, *Enterococcus faecium*, *Escherichia coli*, *Klebsiella pneumoniae*, and *Pseudomonas aeruginosa*) directly from blood within 3 to 5 hours at a level of sensitivity at or above that of conventional blood culture (BC) systems (7). The current CE-marked panel incorporates *Acinetobacter baumannii* as a sixth detectable organism. Building upon this technology, the T2Resistance (T2R) Panel was developed to rapidly detect bacterial genes encoding resistance molecules directly from the bloodstream also within 3 to 5 hours.

T2R Panel is performed on the T2Dx device with T2 Magnetic Resonance (T2MR) detection, detects 13 resistance genes from Gram-positive (*vanA/B*, *mec*A/*mec*C) and Gram-negative (*bla_KPC_*, *bla_NDM_/bla_/IMP_/bla_VIM_*, *bla_CTXM-14/15_*, *bla_AmpC_*, and *bla_OXA_*) bacterial pathogens that commonly cause BSIs (8). As the T2R Panel can identify these resistance genes within 3 to 5 hours from the blood sample receipt within the clinical laboratory, the potential impact on early detection of resistant bacterial pathogens causing sepsis is important. Little is known, however, about the T2R Panel in clinical practice.

We therefore conducted a prospective pilot study of the T2R Panel in two major medical centers, Perugia General Hospital (PGH), Perugia, Italy, and Evangelismos General Hospital (EGH), Athens, Greece, over 5 months. The objective of this study was to evaluate the sensitivity and time of identification of the T2R Panel, for the detection of resistance genes in patients with resistant BSIs in comparison to those of BCs and conventional clinical microbiological methods.

## MATERIALS AND METHODS

### Institutions

The study was conducted from November 2019 through February 2020 in the ICU of EGH and from October 2019 through February 2020 at PGH. The EGH has a 30-bed university ICU in a 943-bed tertiary-care hospital, which admits critically ill adult medical, surgical, and trauma patients. EGH, together with other hospitals in Athens central area covers a population of approximately 643,000. The PGH is an 800-bed tertiary-care hospital, that serves a population of approximately 200,000 people and admits adult and pediatric, medical and surgical, immunocompetent, and immunocompromised patients. Patients at PGH were evaluated from different wards, including two intensive care units (ICUs): a 13-bed ICU, admitting critically ill adult medical, surgical, and trauma patients and an 8-bed ICU admitting post-cardiosurgical patients.

### Inclusion criteria

Patients with a clinical suspicion of sepsis or septic shock (9), in which the attending physician requested BCs, were eligible for enrollment into the study. Patients could be included in the study more than once, provided that the second sample was obtained more than 14 days from initial enrollment.

### Exclusion criteria

The following exclusion criteria were applied in the study: patients who were participants in other studies or had participated in randomized controlled trials during the preceding month; patients who were to be discharged from ICU within the next 24 hours; patients in whom limited treatment was applied; patients younger than 18 years of age; and inability to obtain informed consent from the patient or next of kin.

### Sampling and testing

#### Sample collection and storage

At the same time, the prescribed blood draw for culture, three additional 4 mL EDTA tubes were collected for study. Whole blood collection was performed from the same peripheral vein/anatomic site as those from BCs. Blood cultures were obtained *via* peripheral venous puncture using a standard sterile technique or from a new central venous catheter immediately after placement and prior to breaking the sterile field that was used for the catheterization.

Three 4 mL samples of whole blood were collected. The first sample (Tube 1) was used for the T2Bacteria Panel (T2B) analysis (7). The T2B test was performed immediately upon receipt. The second sample (Tube 2) was indicated for the T2R Panel, classified as Research Use Only, and run sequentially after T2Bacteria. The T2R Panel was run immediately after the T2B Panel due to software design. Finally, a third sample (Tube 3) was frozen (−80°C) and used to repeat the test if the result was invalid or incongruent with respect to the gold standard. If the test was to be repeated, the last result for the final analysis was used. If the second and third samples were not used, they were sent to T2Biosystems as a backup for any confirmation tests.

Both institutions had their instruments. For Perugia, T2 samples were processed by the Microbiology Laboratory. Samples, delivered to the Clinical Microbiology laboratory during the operating time, were processed immediately according to the manufacturer’s instructions. Operating time is from 08:00 a.m. to 08:00 p.m., Monday to Friday, and from 08:00 a.m. to 02:00 p.m. on Saturdays, Sundays, and holidays. For EGH, the T2 MR platform was used as a point of care microbiology assay by ICU-trained staff and operated 24/7 Outside laboratory hours, a Gram stain was communicated and identification and antimicrobial susceptibility testing (AST) were set up after 18-hour incubation and final results were reported the following day.

#### Standard procedures for blood cultures, identification, and AST)

For EVH, three BC bottles were filled with 10 mL of blood each for aerobic, anaerobic, and fungal pathogens, while 4 mL of venous blood was collected in Vacutainer tubes containing 0.129 M (3.8%) trisodium citrate for the T2MR assays. Samples were obtained from patients with newly identified sepsis before any new antibiotics were introduced. All BCs at EGH were processed according to standard laboratory protocols. The continuous monitoring BC system BACTEC 9240 (Becton–Dickinson Sparks, MD, USA) was used for a 5-day incubation protocol. Species identification and susceptibility testing of the blood isolates by minimum inhibitory concentrations to different antimicrobial agents were performed by the VITEK2 system (bioMERIEUX, Marcy l’Etoile, France), and interpreted according to the European Committee on Antimicrobial Susceptibility Testing (EUCAST) standards (10). In cases of coagulase-negative staphylococcal positive cultures, standard criteria for detection of contamination were applied (11). Detection of carbapenemase resistance among BC isolates (*Enterobacterales*, *Pseudomonas aeruginosa*) was performed by NG-Test® CARBA-5 (NG Biotech, Guipry, France), for the five most prevalent carbapenemase families (NDM, IMP, VIM, OXA-48, and KPC). No molecular methods were used at EHG for *AmpC*; only the antibiogram was available for susceptibility testing. The minimum inhibitory concentration (MIC) interpretations, NG-Test CARBA-5 results, and their matching to resistance genes included in the T2R Panel are shown in Table S1. At EGH T2MR assays were performed by the ICU physicians who received the same training and quality control monitoring as laboratory staff, according to the manufacturer’s instructions for use.

For each patient included in the study at PGH, BCs were collected into two BACTEC Plus Aerobic/F and two BACTEC Lytic/10 Anaerobic/F bottles and sent immediately to the microbiology laboratory for incubation in the BD BACTEC FX instrument (Becton Dickinson, Sparks, MD, USA) (12). Three 4 mL samples of whole blood also were collected from the same venipuncture. All mono-microbial positive BCs were processed using the BD Kiestra Work Cell Automation system using automated subculture on solid media and digital imaging following 8 hours of incubation, subsequent species identification, and AST (13) with final results reported the following day during regular laboratory hours. The genes *mec*A/*mec*C were detected by XpertMRSA (Cepheid, Sunnyvale, CA) and AST [cefoxitin disk test (bioMerieux)] plus oxacillin MIC (broth microdilution, MERLIN Diagnostika GmbH); *van*A/*van*B were detected by Xpert *van*A/*van*B (Cepheid) and AST [vancomycin and teicoplanin MIC (broth microdilution, MERLIN Diagnostika GmbH)]. CTX-M 14/15 were detected by immunochromatographic assay lateral flow test (NG Biotech) and extended-beta-lactamase (ESBL) confirmatory testing using the combination disk diffusion test with ceftazidime ± clavulanic acid and cefotaxime ± clavulanic acid. Genes encoding IMP, VIM, NDM, OXA-48, and KPC were detected by Xpert® Carba-R (Cepheid). AmpC was detected phenotypically based on a disk diffusion test. Carbapenem resistance was confirmed by AST (broth microdilution, MERLIN Diagnostika GmbH) and a carbapenemase inhibition disk diffusion test, as described (14). All patients at EGH and 92.3% (24/26) were receiving empiric therapy at PGH at the time of T2 sampling.

### Definitions

Bloodstream infections were classified based on previously established criteria (15, 16). Proven BSIs were defined as a T2B positive and/or positive BC using a concurrently drawn specimen. An infection was considered a Probable BSI if the T2B result was positive and BC negative, and the T2B-detected organism was isolated within 15 days from a clinical BC specimen collected at a different time or from another site. An infection was considered a possible BSI if a negative BC but a positive T2B with supporting clinical evidence of infection or infection treatment for suspected resistant infection.

### T2Resistance panel

The T2R Panel is an *in vitro* diagnostic medical device that operates on the T2Dx instrument and detects bacterial genes, which are commonly associated with resistance to antibacterial agents. The Panel consists of seven detection channels and one internal control channel. The Panel of genetic markers consists of seven classes that are listed in Table 1.

**TABLE 1:** Genetic markers detected by the T2Resistance panel

Genetic biomarker groups	Specific genes detected
*bla*_KPC_	*bla*_KPC_
*bla*_CTX-M_	*bla*_CTX-M 14/15_
Metallo*-*β-lactamase	*bla*_NDM_/*bla*_VIM_/*bla*_IMP_
*bla*_OXA-48_	*bla*_OXA-48_ group
*van*	*vanA*/*vanB*
*mec*	*mec*A/*mec*C
*AmpC*	AmpC (*bla*_CMY_/*bla*_DHA_)
	

The T2R Panel is performed using a 4 mL EDTA blood sample. The T2R Panel runs on the T2Dx instrument that qualitatively detects the 13 resistance genes (Table 1). The T2Dx instrument is a fully automated multiplex instrument that utilizes T2 magnetic resonance detection in a self-contained benchtop system. Briefly, the 4 mL EDTA blood sample is treated with a detergent to selectively lyse red blood cells. The bacterial pathogen cells are then concentrated *via* centrifugation and the supernatant is removed. Following concentration, a synthetic DNA internal control is added, and the bacterial cells and other cellular debris are mechanically disrupted to release the target DNA. This lysate is then amplified by a multiplexed PCR method. The resulting amplified lysate is hybridized with superparamagnetic particles coated with probes designed to bind the amplicon of each target gene detected by the panel. The amplified product is detected by amplicon-induced agglomeration of superparamagnetic particles and T2 magnetic resonance (15).

All processing of samples occurs on the instrument, operators are required to only add the patient blood sample to the cartridge and load this into the T2Dx device. Results are qualitative and are reported as target detected, target not detected, or invalid. There is no indeterminate result with the T2B and T2R Panels.

### Specificity testing

To determine the specificity of the T2R Panel, pathogen-negative blood samples were studied as a component of analytical specificity testing. This study evaluated 100 pathogen-negative blood samples from 10 unique healthy donors each of whom contributed one unit of whole blood.

### Data collection

For each patient, the following data were collected: site of infection, results of BC, susceptibility phenotype or genotype, empirical antimicrobial therapy being administered during T2 collection and directed therapy changes performed after T2 results, date and time of sample collection for BC and T2, and date and time for susceptibility phenotype. Interventions related to results from T2 reports, pathogens recovered from blood, and other sites before or after T2 was performed, for a time interval of 2 weeks were also documented. Times related to the T2 analysis, including the time of introduction of the sample into the machine and the time of the result, were provided by the instrument and recorded in the database.

### Statistical analysis

Differences between time to identification by BCs versus T2B results and BCs versus T2R results for each site and for the combined sites were analyzed by the Kruskal-Wallis test [nonparametric analysis of variance (ANOVA)]. Dunn test with Holm adjustment was used for post hoc adjustment for multiple comparisons. Values were presented as boxplots with median, lower quartile, and upper quartile medians. Differences in ordinal variables were analyzed by 95% confidence intervals and the Mann-Whitney U test. Statistical significance was defined as *P* < 0.05. Sensitivity was determined by the ratio of true positives/(true positives + false negatives) and specificity by the ratio of true negatives/(true negatives + false positives). Analysis was performed using R version 4.2.3 (www.r-project.org/) and GraphPad Prism (version 10.0.2) in Boston, MA.

## RESULTS

### Enrollment

In all, 33 patients were enrolled in the study at the EGH and 26 patients were enrolled at PGH over the course of the study for a total of 59 patients from October 2019 to February 2020. Among the 26 patients enrolled at PGH, 15 were males and 11 were females with a mean age of 57.7 years ± 25.27 (SD), a median of 63.5 years [interquartile range (IQR): 25%–75%], and range of 53–76 years. Among the 33 patients enrolled at EGH, 19 were males with a mean age of 59.9 years ± 17.98 (SD), a median of 63 years (IQR: 25%–75%), and range of 17–87 years. Concomitant conditions assessed by the patient’s care team included septic shock, sepsis, fever and hematological malignancy, acute respiratory distress syndrome (ARDS), pneumonia, intraabdominal infection and/or peritonitis, meningitis, and trauma (Table 2).

**TABLE 2:** Underlying conditions of 59 patients enrolled in the T2Resistance Pilot Study

Condition	Number (%)
Septic shock	19 (32)
Sepsis	11 (19)
Fever and hematological malignancy	11 (19)
Pneumonia	9 (15)
Intracerebral hemorrhage, subarachnoid hemorrhage, or increased intracranial pressure	6 (10)
Trauma	5 (8)
Meningitis or encephalitis	4 (7)
Cardiogenic shock and other cardiovascular diseases	4 (7)
Autoimmune diseases	4 (7)
Acute respiratory distress syndrome (ARDS)	3 (5)
Pneumonia	2 (3)
Intraabdominal infection and/or peritonitis	2 (3)
Endocarditis	2 (3)
Urosepsis	2 (3)
Chronic obstructive pulmonary disease (COPD)	2 (3)
Cardiogenic shock	1 (2)

### Bacteria identified by T2Bacteria

T2Bacteria results were positive in 42.4% (25 of 59) of patients, with identification of 33 bacteria. Overall, pathogens identified by T2B, included: *S. aureus* (*n* = 3), *K. pneumoniae* (*n* = 14), *A. baumannii* (*n* = 11), *P. aeruginosa* (*n* = 2), and *E. coli* (*n* = 3). No *E. faecium* targets were identified. Polymicrobial infections were identified in eight patient cases. Six cases from *K. pneumoniae* and *A. baumannii,* one case from *S. aureus* and *K. pneumoniae,* and one case from *A. baumannii* and *P. aeruginosa*. The T2B panel covered 86% of bacterial pathogens causing BSIs in this patient population. BCs and T2B results (T2B+/BC+, T2B-/BC-) were concordant in 74.6% (50/67) of results. Among the T2B+/BC- cases, a probable BSI was confirmed in 12/16 (75%) of cases through analysis of additional microbiological evidence including BCs and cultures obtained from other infected sites within 15 days of the original T2B blood draw. Four additional possible BSIs were confirmed through clinical and microbiological assessment. BC failed to identify 14 organisms identified by T2B. The performance of T2B in diagnosing BSIs caused by targeted bacteria is further summarized in Table 3. The per-assay sensitivity and specificity of T2B for proven BSIs were 94% (95% CI, 72.7%–99.9%) and 95% (95% CI, 92.4%–97.3%), respectively. When probable and possible BSIs were also considered, the per-assay sensitivity and specificity of T2B increased to 97% (95% CI, 84.7%–99.9%) and 100% (95% CI, 86.8%–100%), respectively. The sensitivity and specificity of the T2B Panel are outlined in Table 4.

**TABLE 3:** Performance of T2Bacteria for diagnosis of proven BSI caused by targeted organisms

T2Channel	Proven BSI (T2+/BC+)	Probable BSI (T2+/BC-)	Possible BSI (T2+/BC-)	False negative (T2-/BC+)	True negative (T2-/BC-)
*E. faecium*	0	0	0	0	59
*S. aureus*	2	1	0	0	56
*K. pneumoniae*	10	2	2	0	45
*A. baumannii*	2	8	1	0	48
*P. aeruginosa*	1	1	0	1	57
*E. coli*	2	0	1	0	56
All	17	12	4	1	321

**TABLE 4:** Sensitivity and specificity for the T2Bacteria panel compared to paired blood cultures and after case adjudication

Specific bacteria detected	Compared to the gold standard (paired blood cultures)				With adjudication^*a*^			
	Sensitivity (95% CI)	Specificity (95% CI)	PPV (95% CI)	NPV (95% CI)	Sensitivity (95% CI)	Specificity (95% CI)	PPV (95% CI)	NPV (95% CI)
*E. faecium*	−	−	−	−	−	−		−
*S. aureus*	100% (15.8–100)	98.3% (90.6 99.9)	66.7% (22.3–93.3)	98.3% (88.5–99.6)	100% (29.2–100)	100% (93.6–100)	100%	100%
*K. pneumoniae*	100% (69.2–100)	91.8% (80.4–97.7)	71.4% (49.4–86.5)	100%	100% (76.8–100)	100% (92.1–100)	100%	100%
*A. baumannii*	100% (99.4–100)	84.2% (72.1–92.5)	50% (35.4–64.6)	100%	100% (71.5–100)	100% (92.6–100)	100%	100%
*P. aeruginosa*	50% (8.4–91.6)	98.3% (90.8–99.9)	50% (8.4–91.6)	98.3 (93.4–99.6)	66.7% (9.4–99.2)	100% (93.7–100)	100%	98.3% (92–99.7)
*E. coli*	100% (15.8–100)	98.2% (90.6–99.9)	66.7% (22.3–93.3)	100%	100% (29.2–100)	100% (93.6–100)	100%	100%
T2Bacteria	94.4% (72.7–99.9)	95.3% (92.4–97.3)	51.5% (39.4–63.5)	99.7% (97.9–99.9)	97.1% (84.7–99.9)	100% (98.9–100)	100%	99.7% (97.9–99.9)

^*a*^ T2Bacteria+/BC- cases were further adjudicated by analysis of additional microbiological evidence including BCs and cultures obtained from other infected sites within 15 days of the original T2Bacteria blood draw.

### Time to identification by T2Bacteria

**Fig 1:**
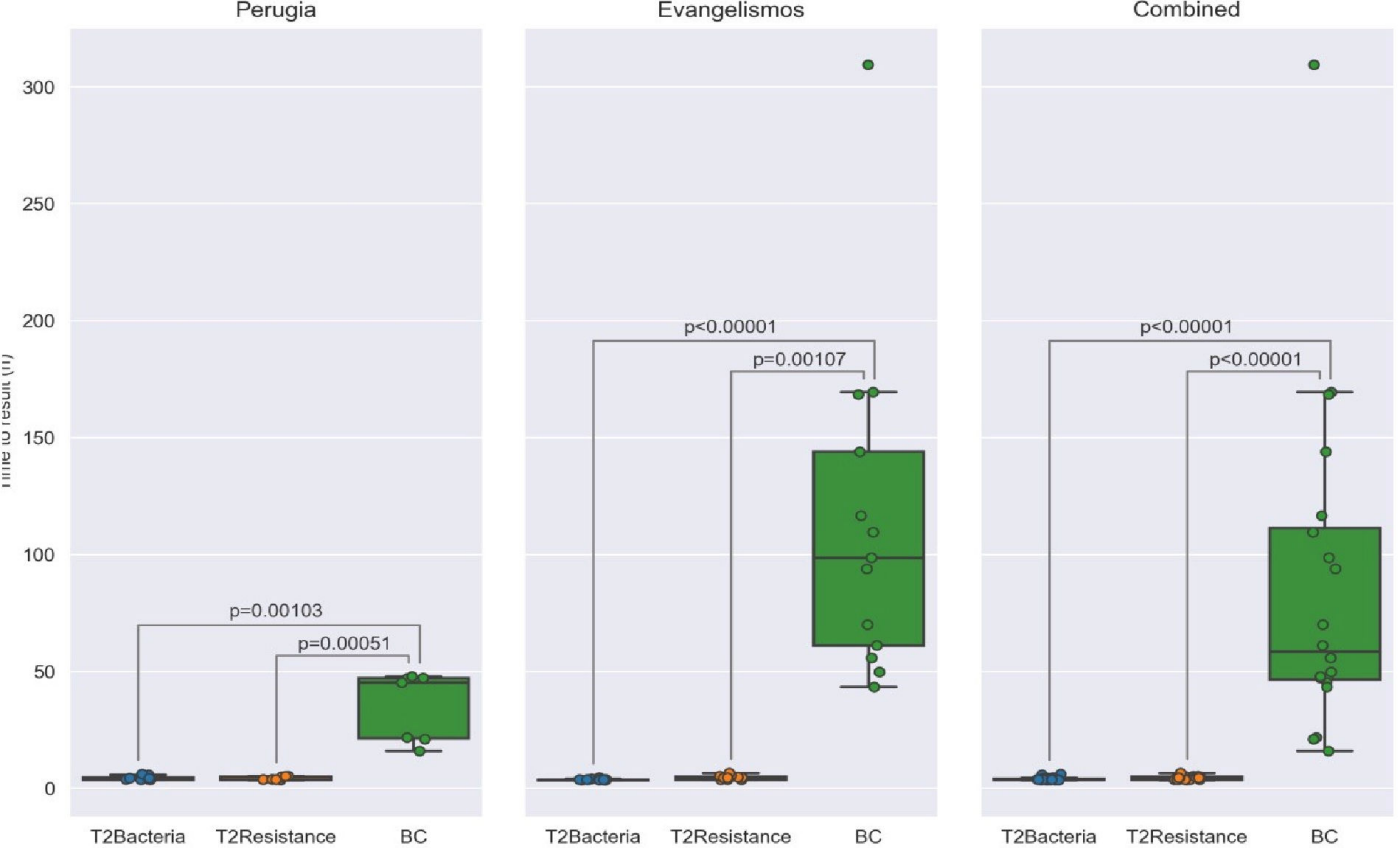
Comparative times to identification from T2Bacteria and T2Resistance versus blood cultures and phenotypic assay and/or molecular assay for both centers and combined. Data are expressed as medians with upper and lower median quartiles. Differences between groups were analyzed by the Kruskal-Wallis test (nonparametric analysis of variance [ANOVA]) and the Dunn test with Holm adjustment for post hoc adjustment for multiple comparisons.

All T2Bacteria tests were performed per the established study protocol. Time to T2B results were measured from the time of sample loading until the result was displayed on T2Dx (Table 5). The median time to species identification by T2B for all patients (*n* = 25) was 3.63 hours (IQR: 3.6–3.93 hours) versus time to final species identification with BCs at 58.34 hours (IQR: 45.51–111.2 hours; *P* < 0.00001 [Fig. 1]). The median time for negative T2B results was 4 ± 0.6 hours compared with a median time to final negative BCs of 154.9 ± 67.9 hours.

**TABLE 5:** Comparative median times to T2Bacteria and T2Resistance identification versus median time to positive blood culture plus phenotypic assay/and/or molecular assay identification for both centers and combined

Site (n)	Time to T2B positive detection (h)			Time to T2R positive detection (h)			Time to BC positive detection (h)			p-value (T2B vs BC)	p-value (T2R vs BC)
	Lower quartile	Median	Upper quartile	Lower quartile	Median	Upper quartile	Lower quartile	Median	Upper quartile		
PGH (*n* = 26)	3.69	3.98	4.61	3.65	3.69	4.91	21.26	45.15	47.03	0.00103	0.00051
	(*n* = 8)			(*n* = 8)			(*n* = 7)				
EGH (*n* = 33)	3.34	3.62	3.65	3.64	4.46	4.91	61.00	98.50	143.80	<0.00001	0.00107
	(*n* = 17)			(*n* = 11)			(*n* = 13)				
Combined (*n* = 59)	3.6	3.63	3.93	3.65	4.4	4.97	45.51	58.34	111.20	<0.00001	<0.00001
	(*n* = 25)			(*n* = 19)			(*n* = 20)				

### Resistance genes identified by T2Resistance

The performance of T2R in diagnosing BSIs caused by resistant pathogens is summarized in Table 6. In all, 25 resistance genes in 19 patients were identified by T2R: *bla*_KPC_ (*n* = 10), *bla*_NDM_*/bla*_IMP_*/bla*_VIM_ (*n* = 5), *bla*_CTXM-14/15_ (*n* = 4), *bla*_AmpC_ (*n* = 2), and *mec*A/*mec*C (*n* = 4). No *vanA*, *vanB*, or *bla*_OXA_ genes were detected.

**TABLE 6:** Detection of all genetic markers identified by T2Resistance (*bla*_CMY_ / *bla*_DHA_, *bla*_CTX-M 14/15_, *bla*_KPC_, *mec*A/*mec*C, *bla*_NDM_ / *bla*_VIM_ / *bla*_IMP_) in comparison to conventional blood cultures

	*bla*_CMY_ / *bla*_DHA_		*bla*_CTX-M 14/15_		*bla*_KPC_		mecA/*mec*C		*bla*_NDM_ / *bla*_VIM_ / *bla*_IMP_	
Site	T2R	BC	T2R	BC	T2R	BC	T2R	BC	T2R	BC
PGH	2	2	4	4	2	2	4	3	0	0
EGH	0	0	0	0	8	6	0	0	5	3
Total positives	2	2	4	4	10	8	4	3	5	3

A detailed patient-level description of the performance of T2R for diagnosis of proven BSI caused by antibiotic-resistant bacteria is delineated in Table 7. The signal for *bla*_KPC_ was detected in 10 patients. Seven of these gene signals were confirmed in paired BCs growing *K. pneumoniae* and one of the seven signals was detected in a patient with polymicrobial bacteremia caused by *K. pneumoniae* and *A. baumannii*. All seven *bla*_KPC_ genes were confirmed with phenotypic or genotypic evidence from the paired BCs. Two of three BC(-) T2R-positive bla_KPC_ signals were adjudicated using secondary microbiological evidence including BCs that grew *K. pneumoniae* harboring a *bla*_KPC_ within 15 days of the original T2R blood draw. The last *bla*_KPC_ signal was adjudicated based on directed antibiotic therapy. T2Resistance failed to identify one *bla*_KPC_ gene in a *K. pneumoniae* identified by both BC and T2B. The blood sample from this assay was not repeated. The final sensitivity for this channel accounts for this missed KPC. No information on variants was available for this isolate.

**TABLE 7:** Patient-based clinical performance of T2Resistance for diagnosis of proven BSI caused by antibiotic-resistant bacteria^*a*^

Patient ID	T2Bacteria	T2Resistance	Paired blood culture	Resistance gene ID or phenotype (AST)	Concordance vs gold standard (blood culture)	Other culture (source)	Other culture resistance gene ID or phenotype (AST)	Concordance vs other cultures resistance gene ID or phenotype (AST)	Additional cases adjudicated based on directed antibiotic therapy
1	KP	NDM/VIM/IMP	KP	No ID	No	KP (BA)	No ID	No	Yes
3	AB	NDM/VIM/IMP	No ID	No ID	No	AB (BA), KP(BC)	VIM	Yes	−
8	KP	KPC	KP	KPC	Yes	−	−	−	−
10	SA, KP	KPC	KP	KPC	Yes	−	−	−	−
13	KP	no ID	KP	KPC	No			No	No
20	KP, AB	KPC, NDM/VIM/IMP	KP, AB	KPC, VIM	Yes	−	−	−	−
21	KP, AB	KPC	KP	KPC	Yes	−	−	−	−
25	KP	KPC, NDM/VIM/IMP	KP	KPC, VIM	Yes	−	−	−	−
26	No ID	KPC	No ID	No ID	No	KP (BE)	KPC	Yes	
27	No ID	NDM/VIM/IMP	No ID	No ID	No	−	−	No	Yes
30	KP, AB	KPC	No ID	No ID	No	KP, AB (BE)	No ID	No	Yes
31	AB	KPC	No ID	No ID	No	KP, AB (BE)	KPC	Yes	
P1	KP	CTX-M	KP	ESBL	Yes	−	−	−	−
P6	KP	KPC, AmpC (CMY/DHA), CTX-M	KP	KPC, ESBL, AmpC-Pheno	Yes	−	−	−	−
P9	SA	mecA/mecC	SA	MRSA	Yes	−	−	−	−
P11	No ID	mecA/mecC	No ID	No ID	No	−	−	No	Yes
P12	KP	mecA/mecC	CoNS	MRCoNS	Yes	−	−	−	−
P13	EC	CTX-M	EC	ESBL	Yes	−	−	−	−
P24	KP, AB	KPC, AmpC (CMY/DHA), CTX-M	KP	KPC, ESBL, AmpC-Pheno	yes	−	−	−	−
P26	No ID	mecA/mecC	CoNS	MRCoNS	Yes	−	−	−	−

^*a*^ AB, *Acinetobacter baumannii*; BA, bronchial aspirate; BE, bronchial expectorant; BC, blood culture; CoNS, coagulase-negative Staphylococcus sp.; EC, *Escherichia coli*; KP, *Klebsiella pneumoniae*; MRCoNS, methicillin-resistant coagulase-negative Staphylococcus sp.; MRSA, methicillin-resistant *Staphylococcus aureus*; SA, *Staphylococcus aureus*.

Five *bla*_NDM_*/bla*_IMP_*/bla*_VIM_ genes were identified by the T2R Panel. Two *bla*_NDM_*/bla*_IMP_*/bla*_VIM_ genes were confirmed using paired BCs that also identified *K. pneumoniae* harboring one of the three target genes. The three additional *bla*_NDM_*/bla*_IMP_*/bla*_VIM_ genes were adjudicated as true infections using secondary BCs obtained within 15 days (*n* = 1) and through clinical and microbiological assessment (*n* = 2).

Four samples that were T2R positive for *bla*_CTXM-14/15_ were confirmed with paired BCs growing *K. pneumoniae* (*n* = 3) and *E. coli* (*n* = 1) harboring the *bla*_CTXM-14/15_ genes. The gene *bla*_AmpC_ was detected in two patients with AST confirmation of AmpC. These two patients, who had bacteremia caused by *Klebsiella pneumoniae* with *bla*_KPC_ and ESBL resistance patterns by conventional BCs, were found by T2B and T2R to have positive signals for *Klebsiella pneumoniae* and *Klebsiella pneumoniae +Acinetobacter baumannii,* both with positive signals for *bla*_KPC_, *bla*_CTXM-14/15_, and *bla*_AmpC_ genes.

In understanding the two T2R-/BC+ events, the first patient was a 67-year-old female with thyrotoxicosis with positive BCs for *Enterococcus faecalis*, *K. pneumoniae*, and *Acinetobacter* sp. The second patient was a 71-year-old female with pneumonia, septic shock, a positive T2B signal for *K. pneumoniae*, and positive BCs for *K. pneumoniae*, but a susceptible phenotype. There were no lower respiratory tract cultures available for this patient.

Lastly, there were four patients with positive signals for mecA/mecC. One of these patients had methicillin-resistant *Staphylococcus aureus* (MRSA) bacteremia from the paired BC. Two patients had bacteremia caused by methicillin-resistant coagulase-negative *Staphylococcus* spp. (*Staphylococcus epidermidis* in one case and *S. epidermidis* plus *S. capitis* in another). The patient with the fourth positive *mec*A/*mec*C signal had no positive BC for *Staphylococcus* spp. but was treated empirically with vancomycin. Two other cases were removed from the final analysis due to invalid results.

### Time to detection by T2R

Among the 59 enrolled patients, 55 were evaluable for assessment of time to detection. Four were excluded from those enrolled at EGH based upon an invalid result (*n* = 2), and insufficient data for assessment of time or BC phenotype (*n* = 2). All T2R tests were performed per the established study protocol. The mean time-to-T2R results was 4.4h ± 0.8 hours for all enrolled patients (*n* = 57) (Fig. 1).

The mean time to positive T2R in PGH was 4.2h ± 0.7 hours, while the mean time to positive BCs with species identification was 34.7 ± 14.4 hours (*P* = 0.001) (Table 7). The mean time to positive T2R in EGH was 4.6 ± 0.9 hours in comparison to that for final reporting of positive BCs with AST of 94.3 ± 47.0 hours (*P* < 0.001).

Moreover, the mean time to a positive result from standard molecular resistance assay (GeneXpert® System) following BCs in 5 patients was 33.7 ± 5.3 hours (*P* = 0.001), from immunochromatographic assay for CTXM in four patients was 27.6 ± 6.9 hours (*P* = 0.008), and from AST in five patients was 53.8 ± 5.8 hours (*P* = 0.004).

### Analytical performance of T2R in comparison to genotypic and phenotypic assays

Overall, per-patient sensitivity and specificity for the T2R panel compared to phenotypic and genotypic detection was 92.3% (95% CI: 64%–99%) and 84% (95% CI: 70%–93.4%) (Table 8). With adjudication, the sensitivity and specificity increased to 94.7% and 97.4%, respectively. Per channel sensitivity was 100% for the *blaCTXM-14/15, blaNDM/bla/IMP/blaVIM, blaAmpC, and mecA/mecC* channels. The sensitivity for *blaKPC was 87.5%*.

**TABLE 8:** Analytical performance of the T2Resistance Panel in comparison to blood cultures

Genetic biomarker groups	Specific genes detected	Blood cultures		Adjudication^*a*^	
		Sensitivity (95% CI)	Specificity (95% CI)	Sensitivity (95% CI)	Specificity (95% CI)
*bla*_KPC_	*bla*_KPC_	87.5% (47.4–99.7)	93.9% (83.1–98.7)	90.9% (58.7–99.8)	97.9% (88.7–99.9)
*bla*_CTX-M_	*bla*_CTX-M 14/15_	100% (39.8–100)	100% (93.3–100)	−	−
Metallo*-*b-lactamase	*bla*_NDM_ / *bla*_VIM_ / *bla*_IMP_	100% (15.8–100)	94.6% (84.9–98.9)	100% (47.8–100)	100% (93.2–100)
*bla*_OXA-48_	*bla*_OXA-48_ group	−	−	−	−
*van*	*vanA*/*vanB*	−	−	−	−
*mec*	*mec*A/*mec*C	100% (29.2–100)	98.1% (90.1–99.5)	100% (39.8–100)	100% (93.3–100)
AmpC	AmpC (*bla*_CMY_/*bla*_DHA_)	100% (15.8–100)	100% (93.5–100)	−	−
All targets		92.3% (63.9–99.8)	84% (69.9–93.4)	94.7% (73.9%–99.8)	97.4% (86.2–99.9)

^*a*^ T2Resistance+/BC- cases were further adjudicated by analysis of additional microbiological evidence including BCs and cultures obtained from other infected sites within 15 days of the original T2Resistance blood draw.

### Specificity testing

The specificity of the T2R Panel was determined by the evaluation of 100 pathogen-negative blood samples from 10 unique healthy donors (Table 9). These studies demonstrated 100% analytical specificity (95% CI: 96.3%–100.0%) for the detection of all genes (*bla*_KPC_, *bla*_CTXM-14/15_, *bla*_NDM_*/bla*_IMP_*/bla*_VIM_, *bla*_AmpC_, _blaOXA_, *vanA/van*B, and *mec*A/*mec*C) tested within the panel.

**TABLE 9:** Specificity of the T2Resistance Panel of 100 samples from 10 donors

Channel	Negative % agreement	
	Specificity (number of samples negative/number of samples tested)	95% CI
*mec*A*/mec*C	100/100 (100%)	96.3%–100.0%
*vanA/van/B*	100/100 (100%)	96.3%–100.0%
KPC	100/100 (100%)	96.3%–100.0%
AmpC	100/100 (100%)	96.3%–100.0%
OXA-48	100/100 (100%)	96.3%–100.0%
NDM/VIM/IMP	100/100 (100%)	96.3%–100.0%
CTX-M14/15	100/100 (100%)	96.3%–100.0%

### Impact on clinically significant antibiotic changes

When monitored for the impact of significant changes in antimicrobial therapy resulting from T2B and T2R results, there were a total of 49 drug-specific antimicrobial interventions in 24 patients, including 17 events of antibiotic additions, and 32 discontinuations. At EGH, there were seven antibiotic additions and 23 events of discontinuation of unnecessary antimicrobial agents in 17 patients. At PGH, there were 19 specific interventions among 7 patients, which consisted of 10 antibiotic additions of antimicrobial agents, and 9 discontinuations. The impact of results from T2R panel on the modification of initial empirical antimicrobial therapy in patients with suspected bloodstream infections and sepsis is described in Table 10 and Table 11.

**TABLE 10:** Clinical impact of results from T2Resistance system on modification of initial empirical antimicrobial therapy in patients with suspected bloodstream infections and sepsis

Antibiotic	Evangelismos (*n* = 17)		Perugia (*n* = 7)		Combined (*n* = 24)	
	New antimicrobials added	Antimicrobial agents discontinued	New antimicrobials added	Antimicrobial agents discontinued	New antimicrobials added	Antimicrobial agents discontinued
Amikacin		1	1	2	**1**	**3**
Ampicillin		2		1	**0**	**3**
Ampicillin/clavulanate		1				**1**
Acyclovir		1			**0**	**1**
Ceftaroline				1	**0**	**1**
Ceftazidime/avibactam	4		3	1	**7**	**1**
Ceftriaxone		1			**0**	**1**
Ciprofloxacin		1				**1**
Cloxacillin	1				**1**	**0**
Colistin		1			**0**	**1**
Daptomycin		1	3	1	**3**	**2**
Linezolid		2			**0**	**2**
Meropenem	2	5	1		**3**	**5**
Piperacillin-tazobactam		1	1	2	**1**	**3**
Rifampin		1			**0**	**1**
Tigecycline		1	1	1	**1**	**2**
Vancomycin		4			**0**	**4**
Total	7	23	10	9	**17**	**32**

**TABLE 11:** Patient-level impact of T2Resistance and T2Bacteria results on modification of initial empirical antimicrobial therapy in seriously ill patients

Patient number	T2Resistance result	T2Bacteria result	Initiation of antimicrobial agent	Appropriate (±)	Discontinuation of antimicrobial agent	Appropriate (±)	Correlative organism and phenotypic resistance (±)
2	ND	EC			Linezolid	+	NA
3	NDM/VIM/IMP	AB	Meropenem	+			Yes
4	ND	ND			Daptomycin	+	NA
5	ND	ND			Colistin, rifampin	+	NA
6	ND	SA	Cloxacillin	+	Ampicillin, acyclovir, ceftriaxone	+	NA
7	ND	ND			Vancomycin	+	NA
8	KPC	KP	Ceftazidime/avibactam	+	Amikacin, vancomycin, tigecycline	+	Yes
10	KPC	SA, KP	Ceftazidime/avibactam	+	Meropenem	+	Yes
12	ND	KP, AB			Ciprofloxacin, ampicillin	+	NA
20	KPC, NDM/VIM/IMP	KP, AB			Vancomycin	+	Yes
21	KPC	KP, AB	Ceftazidime/avibactam	+	Meropenem	+	Yes
22	ND	KP, AB			Meropenem	+	NA
23	ND	ND			Meropenem, vancomycin	+	NA
26	KPC	ND			Linezolid	+	Yes
27	NDM/VIM/IMP	ND			Piperacillin/tazobactam	+	Yes
28	ND	ND	Meropenem	+	Amoxicillin/clavulanate	NA	NA
30	KPC	KP, AB	Ceftazidime/avibactam	+	Meropenem	+	Yes
P1	CTX-M	KP	Ceftazidime/avibactam, daptomycin	+	Amikacin, piperacillin/ tazobactam	+	Yes
P13	CTX-M	EC	Piperacillin/tazobactam	+			Yes
P19	ND	ND			Amikacin	+	NA
P22	ND	ND	Daptomycin	+	Tigecycline	NA	NA
P23	ND	EC	Tigecycline, meropenem	+	Daptomycin, ceftazidime/avibactam, piperacillin/tazobactam	+	NA
P24	KPC, CTX-M, AmpC	KP, AB	Amikacin, ceftazidime/ avibactam	+	Ampicillin, ceftaroline	+	Yes
P26	mecA/C	ND	Daptomycin, ceftazidime/avibactam	+		+	Yes

## DISCUSSION

Early treatment of resistant bacterial BSIs improves outcomes (9, 17). The therapeutic implications of the rapid turnaround time of BC independent molecular tests like T2B and T2R for clinicians caring for septic patients are important and potentially life-saving. This two-center prospective pilot study demonstrated significantly more rapid time to identification of commonly encountered bacteria and genes encoding resistance mechanisms which are commonly encountered in bacterial causes of BSIs and sepsis.

In addition to early recognition of infection, appropriate antimicrobial therapy is known to be crucial for patient survival. Empirical antibacterial therapy for patients with clinical evidence for sepsis commonly consists of an extended spectrum penicillin, such as piperacillin/tazobactam plus vancomycin or an anti-pseudomonal cephalosporin plus vancomycin. Such regimens provide a broad antibacterial spectrum against Gram-negative bacteria and vancomycin-susceptible Gram-positive bacteria. Prior studies of rapid susceptibility testing have depended upon BC-based technology and have not identified genes encoding resistance mechanisms within 3–5 hours of analysis from whole blood.

There is a critical need for rapid diagnostics for patients suffering from sepsis, particularly in the setting of antimicrobial resistance (9, 17–19). In the current study, clinicians were able to intervene in 42% (24/57) of patient cases to optimize therapy immediately after receiving T2R results. In 36% (16/44), T2R facilitated therapeutic antibiotic additions indicating that even broad empiric therapy may miss some potential resistant pathogens. Underscoring the utility of the T2R platform in managing cases of suspected sepsis, the patient population in this study was critically ill with conditions including septic shock, sepsis, hematological malignancies, ARDS, pneumonia, intraabdominal infection, and/or peritonitis.

We also observed that the T2R Panel had a substantial impact on the early modification of the treatment of Gram-negative bacteremia. Early identification of a *K. pneumoniae* with a KPC report would warrant a change to a KPC-active beta-lactam/beta-lactamase inhibitor combination. In the present study, clinicians were able to initiate ceftazidime/avibactam in six patients in response to positive T2R results for blaKPC. Similarly, as carbapenems are generally recommended for the treatment of serious infections, including BSIs and sepsis, early detection of AmpC would warrant initiation of a carbapenem, pending more clinical and phenotypic data. Carbapenems are also recommended for the treatment of serious infections caused by organisms encoding CTX-M14 *and* CTX-M15 genes. Early detection of these genes would also prompt initiation of carbapenem therapy. Detection of genes encoding NDM, VIM, and IMP would warrant treatment of suspected bacterial pathogens with agents like ceftazidime/avibactam combined with aztreonam or cefiderocol monotherapy, which have broad spectrum activity against difficult-to-treat Gram-negative bacteria, which are resistant to front-line antibiotics used for initial empirical antibacterial therapy in patients with sepsis.

For the treatment of Gram-positive bacteremia and sepsis, rapid identification of *vanA/vanB* would allow for the initiation of daptomycin or linezolid for the treatment of vancomycin-resistant *Enterococcus faecium* (VRE). Although this study did not detect *vanA/vanB* by T2R or *E. faecium* by T2B or in BCs, the therapeutic implications are important for early targeted treatment. Finally, rapid detection of *mec*A*/mec*C would allow continuation of vancomycin as empirical therapy, further investigation of source, removal of vascular catheters, and de-escalation of Gram-negative antimicrobial therapy depending upon the clinical status of the patient.

The T2R system builds upon the time-tested technology of the T2B Panel developed by T2 Biosystems as the only FDA-cleared test to identify sepsis-causing bacterial pathogens directly from whole blood without the need for growth of organisms in BC and within a rapid turnaround time of 3–5 hours (7). The original pivotal study of T2B demonstrated the superiority of the T2 magnetic resonance technology in detection of targeted bacteria (*Escherichia coli*, *Acinetobacter baumannii Staphylococcus aureus*, *Klebsiella pneumoniae*, *Pseudomonas aeruginosa*, and *Enterococcus faecium*), where BCs were positive in 39 (3%) of 1,427 patients and T2B results were positive in 181 (13%) of 1427 patients (7). Following this clinical trial, Giannella *et al*. conducted a systematic review of randomized trials and observational controlled studies comparing T2MR systems and BCs (20). Employing an inverse variance meta-analysis model, the investigators demonstrated that utilization of results from the T2MR platform yielded more rapid time to detection, transition to targeted microbial therapy, and reduction of empirical therapy, as well as decreased length of stay in intensive care units and hospitalizations. Mortality rates were comparable.

Paggi and colleagues further investigated the impact of T2B results on patient management (21). T2B in this study again proved to be significantly more sensitive in the detection of the targeted bacterial causes of sepsis (100%, 95% CI: 86.3–100.0) versus that of BCs (54.8%, 95% CI: 36.0–72.7). Management based upon T2B resulted in significantly reduced time of patients receiving empirical therapy and greater time on pathogen-targeted therapy.

With the advent of patients suffering from severe COVID-19, the need for rapid diagnosis of sepsis and accurate detection of resistant pathogens causing BSIs has become particularly paramount (22). An Austrian-based study (23), which investigated the expanded T2B Panel (*Escherichia coli*, *Staphylococcus aureus*, *Klebsiella pneumoniae*, *Pseudomonas aeruginosa*, *Enterococcus faecium,* and *Acinetobacter baumannii*) and T2Candida in patients with severe COVID-19 identified T2MR detected targeted bacterial pathogens in 9 (10.6%) of 85 samples in comparison to parallel BCs detecting in 3 (3.5%) of 85 samples. T2Candida also was more sensitive in detection of candidemia than BCs [7 (8.2%) of 85 versus 1 (1.2%) of 85, respectively]. The time to detection of the etiological agent of BSIs also was significantly reduced (4.5 for T2 versus 52.5 hours for BCs). In the current study, we observed a sensitivity for T2B of 94.4%, which improved to 97.1% after adjudication.

When analyzing for detection of a biomarker using a more sensitive assay than that of the gold standard, the challenge becomes one of documenting specificity. As a solution to these analytical challenges, two measures were implemented. First, a study of 100 samples of pathogen-free blood from 10 volunteers was conducted. That study found no false positives, yielding an analytical specificity of 100% with 95% CI of 96.3%–100.0%. The second approach is to assess the T2R+/BC- cases through adjudication of confirmatory clinical and microbiological data supporting the T2R+ results. The specificity in this study after adjudication was 100%.

The past decade has witnessed major advances of molecular diagnostic tools in clinical microbiology laboratories (24). While key advances have been achieved in rapid identification and detection of resistance-encoding genes of bacterial pathogens recovered from positive BCs, T2MR technology has been distinctive in being the only currently FDA-licensed technology for rapid identification of bacterial pathogens and *Candida* spp. directly from the bloodstream. Adding to this technology is the rapid detection of resistance-encoding genes using the T2R Panel directly from bacteria-causing BSIs. Building upon the initial observations of De Angelis and colleagues, which detected carbapenem resistance genes in 11 (84.6%) of 13 whole blood samples from patients with culture-positive BSIs (16), this report expands the patient population studied and demonstrates the direct clinical utility of the data.

The presence of discordant results between T2R and standard phenotypic/genotypic methods may be based upon differences in the sensitivity of recovery of pathogens in the bloodstream. Previous studies have found T2B to be more sensitive than conventional BCs in detecting blood-borne pathogens when such cases are analyzed for other sources, such as pneumonia or intra-abdominal infections (7, 20, 21). Given the high analytical specificity of the T2R Panel, the probability of false-positive results seems less likely than greater the likelihood of detecting of the resistance genes of circulating pathogens that have not been detected by conventional BCs. In the current study, we observed a per-patient sensitivity and specificity of 92.3% and 84.1%, respectively, which increased to 94.7% and 97.4%, respectively, after adjudication.

Most patients were already receiving empirical antibacterial therapy at the time of simultaneous T2 and BC sampling. Although BC may be negative from the antimicrobial effect of empirically administered antibiotics, T2B and T2R may still be positive based on the presence of nonviable, nonproliferating, or latent bacteria. The clearance of BCs with the persistence of a positive T2B signal carries potentially important clinical implications of poorer outcomes consistent with ongoing infections (25).

This study has several limitations. First, the number of patients enrolled and centers participating are relatively small in comparison to those of the large definitive study of T2B (7). Second, time to test result availability for T2R was limited due to preliminary software that did not permit simultaneous testing with both T2B and T2R. The currently deployed instruments for a clinical practice able to simultaneously run both T2B and T2R. In addition, because this was a preliminary study, there was minimal lead-in time for clinicians to become comfortable with this novel test, hence potentially affecting their comfort levels with therapeutic changes. Third, two samples in this study were considered to be invalid due to inadequate whole blood volumes or other operational interreferences. In addition, access to microbial samples for patients with pulmonary and other sources of sepsis was limited. Nonetheless, this pilot study demonstrates proof of concept that the T2R Panel can detect commonly encountered bacterial genes encoding resistance mechanisms in patients with BSIs and sepsis at significantly more rapid turnaround times than those of conventional culture-based phenotypic and genotypic methods. In addition, the study also demonstrated that clinicians were able to intervene for their patients based upon these results. Moreover, this study provided a conceptual framework for the development of the larger study of T2R that is ongoing (NCT05231187).

The addition of the new T2R Panel expands the clinical utility of the T2MR platform for rapid identification of resistant bacterial pathogens for modification of empirical antibacterial therapy of organisms carrying resistance genes encoding KPC, CTX-M-14/15, AmpC, NDM/IMP/VIM, *mec*A/*mec*C, and *vanA/*v*anB*, as well as allowing for possible de-escalation of Gram-negative coverage for patients with detected *mec*A/*mec*C.

## AFTER EPUB

*[This article was published on 8 March 2024 with errors in Results and Table 10. The errors were corrected in the current version, posted on 27 March 2024.]*

## SUPPLEMENTAL MATERIAL

Table S1 jcm.01296-23-s0001.docx Determination of resistance by phenotypic laboratory tests and correlation with the T2Resistance panel.

ASM does not own the copyrights to Supplemental Material that may be linked to, or accessed through, an article. The authors have granted ASM a non-exclusive, world-wide license to publish the Supplemental Material files. Please contact the corresponding author directly for reuse.
