# Supplementary material for: Prospective observational pilot study of the T2Resistance panel in the T2Dx system for detection of resistance genes in bacterial bloodstream infections
Source: J Clin Microbiol. 2024 Mar 8;62(4):e01296-23. doi: 10.1128/jcm.01296-23 (PMC11005387; doi:10.1128/jcm.01296-23)
Supplement: Table S1 — Determination of resistance by phenotypic laboratory tests and correlation with the T2Resistance panel. [file jcm.01296-23-s0001.docx]

**Supplemental Table 1.**

**Determination of Resistance by Phenotypic Laboratory Tests and Correlation with the T2Resistance Panel**

|  | ***Pseudomonas aeruginosa*** | | ***Klebsiella pneumoniae*** | | |
| --- | --- | --- | --- | --- | --- |
|  | **TND** | **NDM/VIM/IMP** | **TND** | **KPC ± CTX-M** | **NDM/VIM/IMP ± KPC^a^** |
| aztreonam | S | I/R | S | R | R |
| ceftazidime | - | - | S | R | R |
| ceftolozane/tazobactam | S | - | - | - | - |
| imipenem | S | R | S | R | R |
| meropenem | S | R | S | R | R |
| ertapenem | - | - | S | - | - |
| piperacillin/tazobactam | S | - | - | - | - |
| ceftazidime/avibactam | - | R | S | S | R |
| cefotaxime | - | - | S | R | R |
| cefepime | - | - | - | - | - |
| ESBL | - | - | Neg (-) | Neg (-) | Neg (-) |
|  | ***Staphylococcus aureus*** | | ***Enterococcus faecium*** | ***Providencia stuartii*** | |
|  | **TND** | ***mec*A/*mec*C** | ***vanA/vanB*** | **AMP-C** | **NDM/VIM/IMP** |
| cefoxitin test | - | + | - | - | - |
| oxacilin | S | R | - | - | - |
| vancomycin | - | - | R | - | - |
| teicoplanin | - | - | R | - | - |
| imipenem | - | - | - | S | R |
| meropenem | - | - | - | S | R |
| ertapenem | - | - | - | - | R |
| piperacillin/tazobactam | - | - | - | S | R |
| cefepime | - | - | - | I | R |

**Table Footnotes:** T2R: T2Resistance assay; TND: target not detected; S: susceptible; R: resistant; I: intermediate; KPC: carbapenemase*-*producing *Klebsiella pneumoniae*; AmpC: ampicillinase C beta-lactamase; VIM: Verona integron-encoded metallo-β-Lactamase; IMP: active-on-imipenem metallo-β-lactamase; NDM: New Delhi metallo-beta-lactamase 1; VanA/B: vancomycin resistance gene; *mecA/C*: methicillin resistance genes; *C*TX-M: Cefotaximase-Munich – Allie

^a^carba 5 immunochromatography was performed to differentiate carbapenemases
